# *Bacillus thuringiensis* Toxins: An Overview of Their Biocidal Activity

**DOI:** 10.3390/toxins6123296

**Published:** 2014-12-11

**Authors:** Leopoldo Palma, Delia Muñoz, Colin Berry, Jesús Murillo, Primitivo Caballero

**Affiliations:** 1 Instituto de Agrobiotecnología, CSIC-UPNA-Gobierno de Navarra, Campus Arrosadía, Mutilva Baja, 31192 Navarra, Spain; E-Mail: leopoldo.palma@unavarra.es; 2 Grupo de Protección Cultivos, Departamento de Producción Agraria, Escuela Técnica Superior de Ingenieros Agrónomos, Universidad Pública de Navarra, Pamplona, 31006 Navarra, Spain; E-Mails: dmunoz@unavarra.es (D.M.); jesus.murillo@unavarra.es (J.M.); 3 Cardiff School of Biosciences, Cardiff University, Park Place, Cardiff CF10 3AT, UK; E-Mail: Berry@cf.ac.uk

**Keywords:** *Bacillus thuringiensis*, Bt biopesticides, toxic activity, Cry toxins, Cyt toxins, Vip toxins, Sip toxins, parasporins

## Abstract

*Bacillus thuringiensis* (Bt) is a Gram positive, spore-forming bacterium that synthesizes parasporal crystalline inclusions containing Cry and Cyt proteins, some of which are toxic against a wide range of insect orders, nematodes and human-cancer cells. These toxins have been successfully used as bioinsecticides against caterpillars, beetles, and flies, including mosquitoes and blackflies. Bt also synthesizes insecticidal proteins during the vegetative growth phase, which are subsequently secreted into the growth medium. These proteins are commonly known as vegetative insecticidal proteins (Vips) and hold insecticidal activity against lepidopteran, coleopteran and some homopteran pests. A less well characterized secretory protein with no amino acid similarity to Vip proteins has shown insecticidal activity against coleopteran pests and is termed Sip (secreted insecticidal protein). Bin-like and ETX_MTX2-family proteins (Pfam PF03318), which share amino acid similarities with mosquitocidal binary (Bin) and Mtx2 toxins, respectively, from *Lysinibacillus sphaericus*, are also produced by some Bt strains. In addition, vast numbers of Bt isolates naturally present in the soil and the phylloplane also synthesize crystal proteins whose biological activity is still unknown. In this review, we provide an updated overview of the known active Bt toxins to date and discuss their activities.

## 1. Introduction

*Bacillus thuringiensis* (Bt) is a ubiquitous Gram-positive, rod-shaped and sporulating bacterium that has been isolated worldwide from a great diversity of ecosystems including soil, water, dead insects, dust from silos, leaves from deciduous trees, diverse conifers, and insectivorous mammals, as well as from human tissues with severe necrosis [1,2,3,4]. Bt strains produce a wide variety of insecticidal proteins active against larvae of very diverse insect orders as well as, in some cases, against species from other phyla. This has led Bt-based products to become the best selling biological insecticides to date [4,5] since the genes encoding insecticidal proteins have been successfully used in novel insecticidal formulations and in the construction of transgenic crops [6].

**Figure 1:**
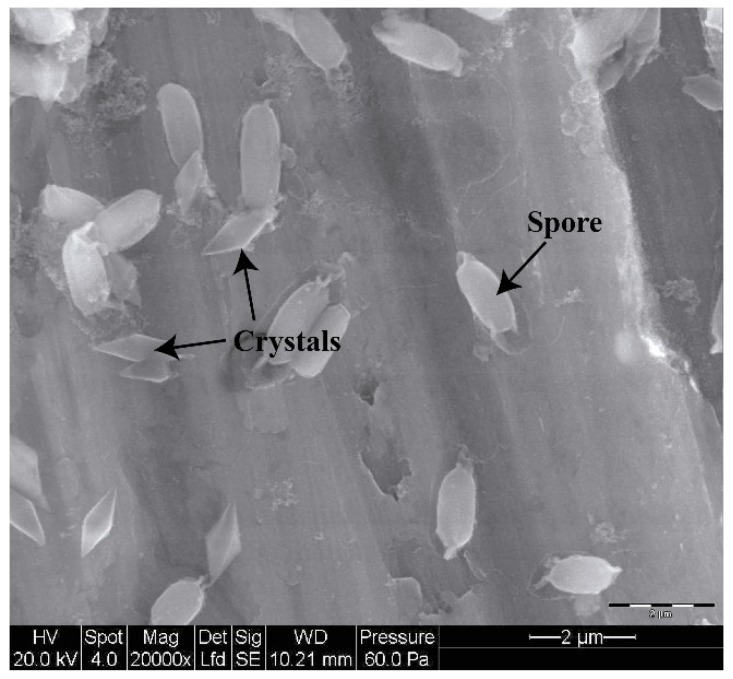
Protein crystals (bipyramidal) mixed with spores from Bt strain H29.3.

Bt strains synthesize Crystal (Cry) and cytolytic (Cyt) toxins, (also known as δ-endotoxins), at the onset of sporulation and during the stationary growth phase as parasporal crystalline inclusions (Figure 1). Once ingested by insects, these crystals are solubilized in the midgut, the toxins are then proteolytically activated by midgut proteases and bind to specific receptors located in the insect cell membrane [5,7], leading to cell disruption and insect death.

In the past decades, more than 700 *cry* gene sequences that code for crystal (Cry) proteins have been identified [1,5,8,9] and large plasmids appear to be the usual location for these genes. While many Cry proteins have useful pesticidal properties and may be exploited for the control of insect pests in agriculture (e.g., [10]) other proteins produced as parasporal crystals by Bt strains have no known invertebrate target and have been termed parasporins. Some of this parasporin group of Cry proteins, such as Cry31A, Cry41A, Cry45A, Cry46A, Cry63A and Cry64A, exhibit strong and specific cytocidal activity against human cancer cells of various origins and have been given the alternative names parasporin-1 (PS1), parasporin-3 (PS3), parasporin-4 (PS4), parasporin-2 (PS2), parasporin-6 (PS6), and parasporin-5 (PS5), respectively [11,12]. Additionally, Bt isolates can also synthesize other insecticidal proteins during the vegetative growth phase; these are subsequently secreted into the culture medium and have been designated as vegetative insecticidal proteins (Vip) [13,14] and the secreted insecticidal protein (Sip) [15]. Vip proteins are classified into four families Vip1, Vip2, Vip3 and Vip4 according to their degree of amino acid similarity. The binary toxin comprising Vip1 and Vip2 proteins [14] and the Sip toxin [15] exhibit insecticidal activity against some coleopterans, whereas Vip3 toxins are toxic against lepidopterans [15]. The host spectrum of the Vip4Aa1 toxin remains to date unknown.

Bt crystal and secreted soluble toxins are highly specific for their hosts and have gained worldwide importance as an alternative to chemical insecticides. The usefulness of these insecticidal proteins has also motivated the search for new Bt isolates from the most diverse habitats in order to identify and characterize new insecticidal proteins with different specificities. Some of these isolates exhibit novel and unexpected toxic activities against organisms other than insects, suggesting a pluripotential nature of some toxins.

## 2. Bt Toxin Nomenclature

**Figure 2:**
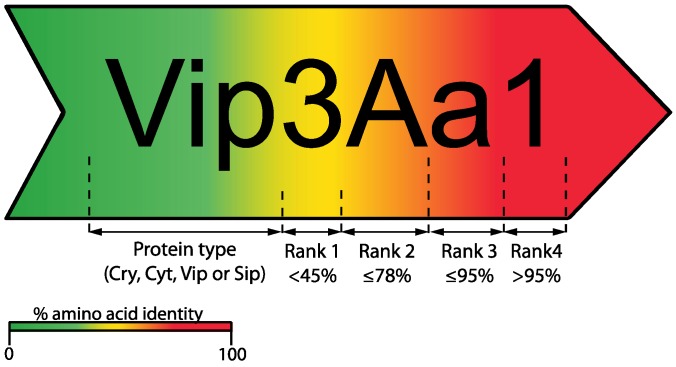
Schematic overview of the current nomenclature system used by the Bt Toxin Nomenclature Committee for δ-endotoxins (Cry and Cyt) and secretable (Vip and Sip) toxins [8]. In this example, numbers indicate different Vip proteins changing rank 1 depending of percentage amino acid similarity (for Vip proteins this rank may change to date among Vip1, Vip2, Vip3 and Vip4). The same rule applies for ranks 2, 3 and 4 assigning a different identification digit/letter.

Since the identification and cloning of the first Bt insecticidal crystal protein gene in 1981 [16], the number of genes coding for novel insecticidal proteins has continuously increased, generating the need for an organized nomenclature system. In the first such system, names for Cry toxins and their corresponding genes included a Roman numeral (primary rank distinction) depending on the insecticidal activity of the crystal protein, namely: CryI for proteins toxic for lepidopterans, CryII for proteins with toxicity against both lepidopterans and dipterans, CryIII for proteins toxic for coleopterans; and CryIV for proteins toxic exclusively for dipterans [1]. However, this system exhibited important complications; for instance, the activity of new toxins had to be assayed against a growing list of insects before the gene and the toxin could be named, some novel homologous proteins were in fact non-toxic as expected, and others (e.g., Cry1I) exhibited dual toxicity against dipteran and lepidopteran species [17]. To avoid these problems, the *Bacillus thuringiensis* Toxin Nomenclature Committee was created and a novel system of classification proposed [8,17]. In this new system, a novel toxin is given a four-rank name depending on its degree of pairwise amino acid identity to previously named toxins; additionally, grouping by this criterion does not imply a similar protein structure, host range or even mode of action. Arabic numbers are used for the first and fourth ranks, and uppercase and lowercase letters are assigned for the second and third ranks, respectively (Figure 2). In this way, proteins sharing less than 45% pairwise identity are assigned a different primary rank (an Arabic number, e.g., Vip1 and Vip2); two proteins sharing less than 78% pairwise identity are assigned a different secondary rank (a capital letter, e.g., Vip3A and Vip3C); proteins sharing less than 95% pairwise identity are assigned a different tertiary rank (a lowercase letter, e.g., Vip3Aa and Vip3Ab); and, finally, to differentiate between proteins sharing more than 95% pairwise identity, a quaternary rank is assigned (an Arabic number, e.g., Vip3Aa1 and Vip3Aa2) [8,17]. However, such quaternary ranks are assigned to each independently sequenced toxin-coding gene; therefore, although some proteins may have different quaternary ranks, they could actually share identical amino acid sequences [8]. This nomenclature system is commonly applied to δ-endotoxins (Cry and Cyt) and secretable (Vip and Sip) Bt toxins.

## 3. Crystal Toxins (δ-endotoxins)

Crystal proteins are formed as parasporal crystalline inclusions during the stationary phase of growth [1,5]. The most widely known are the δ-endotoxins, including Cry and Cyt toxins. Cry proteins can be sub-divided into groups according to their homology and molecular structure. As mentioned above, crystal toxins with no known invertebrate activity have both Cry and Ps designations [12]. These proteins belong to either the three-domain (Cry31 or Ps1, Cry41 or Ps3, Cry63 or Ps6) or the ETX_MTX2 family proteins (Cry45 or Ps4, Cry46 or Ps2, Cry64 or Ps5) and exhibit strong and specific cytocidal activity against human-cancer cell lines (upon protease activation) [11].

The Cyt proteins constitute a smaller, distinct group of crystal proteins with insecticidal activity against several dipteran larvae, particularly mosquitoes and black flies [7,18,19,20,21,22]; additionally, some Cyt toxins are capable of synergizing the insecticidal activity of other Bt proteins [22,23].

### 3.1. Cry Toxins

The naming of a protein as a Cry toxin derives from the fact that it forms a parasporal crystal. As a result, Cry toxins do not belong to a single, homologous family of proteins but, instead, include a number of unrelated lineages. The largest group comprises the well-known three-domain Cry proteins, whereas other Cry toxins belong to distinct protein families e.g., binary Bin- and ETX_MTX2-like toxins produced by *Lysinibacillus* *sphaericus* (Ls, formerly known as *Bacillus sphaericus*) [21,24] as described below.

**Figure 3:**
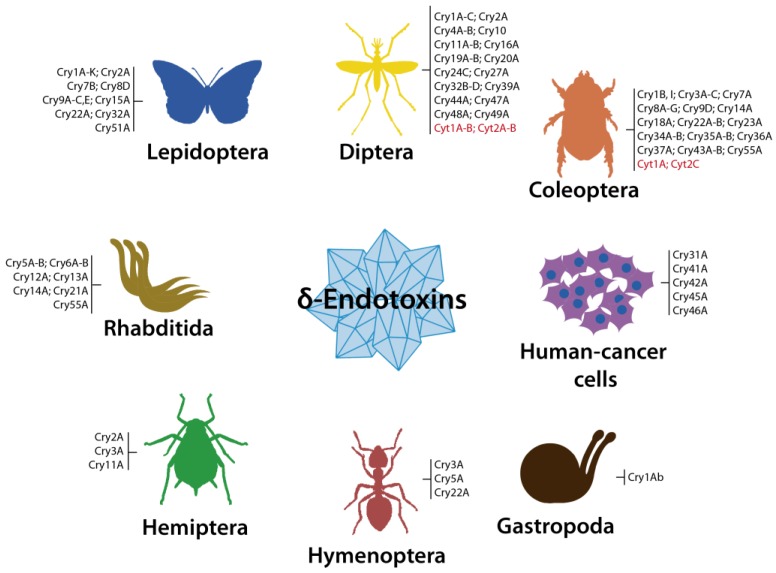
Summarized view showing the known host spectrum of Bt δ-endotoxins (Cry and Cyt) [9,26]. Cry1A-C (separated by hyphen) indicates a group of C3y1A, Cry1B and Cry1C toxins. Cry1B, I (separated by colon) indicates different Cry1B and Cry1I toxins. Semicolons separate groups or individual toxins. Cyt toxins are in red.

Currently, the Cry proteins constitute the largest group of insecticidal proteins produced by species of *Bacillus*. To date, the Bt Toxin Nomenclature Committee [8] has classified 73 different types (Cry1 to Cry73) of Cry proteins, including three-domain and ETX_MTX2 family proteins from Bt and Ls, with individual toxins showing well documented toxicity against lepidopterans, coleopterans, hemipterans, dipterans, nematodes (human and animal parasites, and free living; Rhabditida) some snails [1,5,9,18,21,25,26,27,28] and/or human-cancer cells of various origins [11,12] (Figure 3).

Some Cry proteins may be responsible for other novel toxic properties. Bt strains B622 and B626, isolated in Japan, produced parasporal crystal proteins exhibiting activity against the human pathogenic protozoan *Trichomonas vaginalis* as well as producing lectin-like effects (agglutination) on rabbit erythrocytes [29]; however, they did not show any insecticidal effect against the lepidopteran *Plutella xylostella* and the dipteran *Culex pipiens molestus* [30]. Additionally, the crystal proteins produced by Bt strains 977 and NRRL HD-522 displayed moderate but specific molluscicidal activity against Chinese *Oncomelia* snails [27,31], the intermediate host of the trematode worm *Schistosoma japonicum*, the cause of endemic schistosomiasis in China and The Philippines [32].

Certain crystal toxins show also undesirable activities, such as the haemolytic activity described for Cry15A toxin [33]. Finally, other Cry toxins have also shown antibacterial activity. This is of great relevance for the Bt community because procedures to evaluate the insecticidal activity of crystal proteins generally involve their expression in *E. coli* to obtain pure proteins, and a potential antibacterial activity might hamper their cloning and/or adequate expression (e.g., the three-domain toxins Cry13A and Cry14A) [34]. Additionally, when supplied exogenously, the *B. thuringiensis* subsp. *israelensis* toxins Cyt1Aa, Cry4Ba and Cry11Aa are bactericidal or bacteriostatic to *E. coli* and three Gram-positive bacterial species, whereas Cry1A, Cry3A and a CryD-like toxin, produced by other Bt subspecies, displayed antibacterial activity upon proteolytic activation against species of the anaerobic Gram-positive genus *Clostridium* and to an archaeal species [18,35,36,37]. Other Cry and Cyt-like sequences have also been reported to be bactericidal [35,36,37].

#### 3.1.1. Three-Domain Cry Toxins

**Figure 4:**
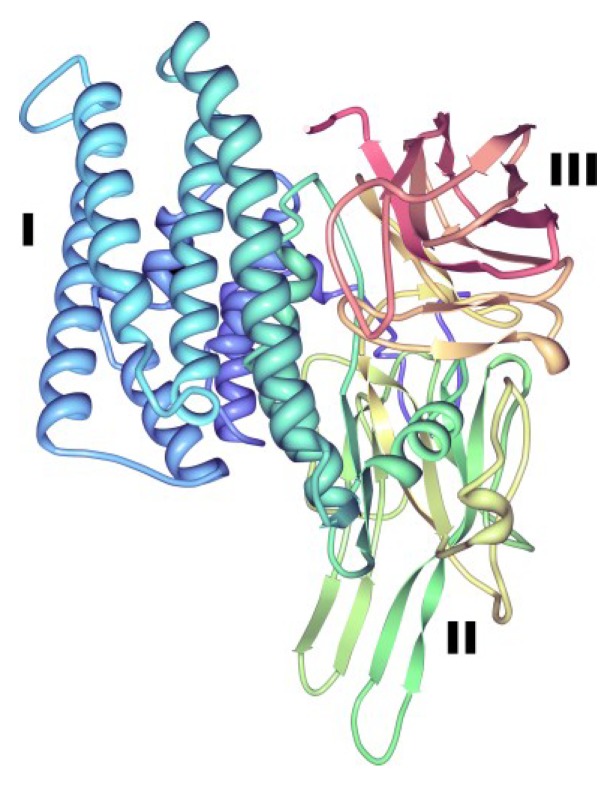
Three-dimensional structure of Cry2Aa toxin. This structure from PDB accession number 1I5P [40], is representative of a three-domain toxin produced by Bt. Roman numerals indicate the typical domains of the three-domain Cry proteins: (**I**), perforating domain; (**II**), central domain, involved in toxin-receptor interactions; (**III**), galactose-binding domain, involved in receptor binding and pore formation [7,21,38,39].

Cry toxins belonging to the three-domain Cry toxin family, display clear differences in their amino acid sequences but all share in common a remarkably similar and conserved three-domain structure [7,21,38,39] (Figure 4).

**Figure 5:**
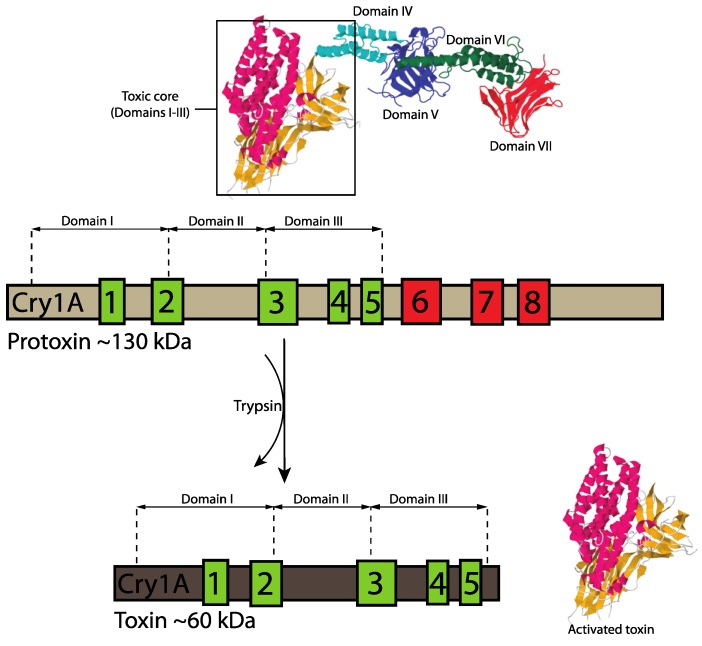
Amino acid conserved blocks (1 to 8) among different three-domain Cry proteins (colored boxes) [5]. Green boxes represent the five conserved amino acid blocks first described by Höfte and Whiteley located in the Cry protein toxic core [1]. Red boxes indicate the three additional conserved amino acid blocks found by Schnepf *et al.* [5]. The protoxin is digested by midgut proteases into a smaller fragment, which is responsible for toxic activity. Three-dimensional structure changes upon proteolytic activation are also depicted for Cry1Ac protoxin: toxic core comprising domains I, II and III (boxed), domain IV (cyan), domain V (blue), domain VI (green), and domain VII (red) [43].

Domain I or perforating domain, located towards the *N* terminus, is constituted by a seven α-helix cluster that is subjected to proteolytic cleavage in all three-domain Cry proteins during toxin activation (Figure 4) and this may be responsible for toxin membrane insertion and pore formation [5,18,41]. Domain II (central or middle domain) consists of three antiparallel β-sheets and plays an important role in toxin-receptor interactions [41,42]. Lastly, domain III (galactose-binding domain), which is proteolytically cleaved in some three-domain Cry proteins, is a two antiparallel β-sheet sandwich that is also involved in receptor binding and pore formation [41]. A recent paper has revealed the structure of the protoxin form of Cry1Ac [43] (PDB accession number 4W8J), demonstrating that the toxin region is already folded into the three-domains while the extended pro-region forms a further 4 domains. Domains IV and VI are alpha helical, whereas domains V and VII have a beta roll topology and in the dimer seen in the packing of the crystals, the toxic region of one monomer is cradled by the pro-region of the other. Although the proCry1Ac used in these studies was mutated to remove 14 of the 16 cysteine residues in order to facilitate the experiments, observation of the natural location of these residues in the protoxin structure gives indications of the likely disulphide cross-linking that stabilizes the natural Bt crystals [44]. Such inter-molecular disulphide bonds are more labile than intra-molecular bonds [45] and are labile under acid and alkaline conditions, explaining the solubilization of Bt toxins in the environment of the insect gut. Multiple-sequence alignments of different Cry toxins also revealed the presence of up to five typical conserved blocks located in the active toxic core of the protoxins (domains I, II and III) which is released after protein activation by midgut proteases [1] and three additional conserved amino acid blocks lying outside this active core and towards the *C*-terminal end of the protoxin (Figure 5). Several of the three-domain protoxins (e.g., Cry3 and Cry11 toxins) lack the extended *C*-terminal region and are, instead, synthesized as shorter protoxins of approximately 70-kDa.

Bt three-domain Cry proteins display toxic activity against insect species of the following orders: Lepidoptera, Diptera, Coleoptera, Hemiptera (low to moderated toxicity for some aphids) and nematodes [9,26].

The mode of action of three-domain crystal proteins has been studied mainly in lepidopteran insects [7]. Three different models have been proposed to explain the mode of action of three-domain Cry toxins: the “classical” model, the sequential binding model and the signaling pathway model [46]. The “classical” model basically proposes that the toxin lyses the midgut epithelial cells of susceptible insects throughout the following steps: (a) crystal inclusion ingestion and dissolution in the alkaline midgut lumen; (b) protoxin (native protein) proteolytic activation that turns the native Cry protein into smaller protease-resistant toxic polypeptides; (c) binding of toxin fragments to specific receptors on the surface of midgut epithelial cells; and (d) formation of non-selective pores permeable to inorganic ions, amino acids and sugars [46,47]. Such pores produce the lysis of epithelial cells and hence midgut disarrangements, leading to insect death. Additionally, spores may colonize, germinate, and replicate in the hemolymph, eventually killing larvae by septicemia [1,2,5]. Although this scheme has been accepted for many years, some details still remain poorly understood (e.g., pore structure and mechanism of pore assembly) [46]. The sequential binding model suggests that Cry toxins, once activated by intestinal proteases, bind to cadherin-like proteins (transmembrane glycoproteins that function as toxin receptors) and undergo a conformational change that favors proteolytic removal of the α-1 helix from domain I and formation of an oligomeric pre-pore structure. Later, binding to a secondary receptor, such as an aminopeptidase, facilitates the insertion of the pre-pore structure into the membrane, leading to cell and insect death [7,39]. In contrast, the signaling-pathway model suggests that the toxic activity is mediated by the specific binding to cadherin receptors, leading to undescribed Mg^2+^-dependent and adenylyl cyclase/protein kinase A signaling pathway that produces necrotic cell death [48]. Vachon *et al.* (2012) have recently reviewed experimental evidence supporting both the sequential binding and the pathway-signaling models [46]. These authors concluded that both models, and more importantly the sequential binding model, are supported by little reliable experimental evidence and that the present available information supports the “classical” model postulating that Cry toxins act by forming pores, although most events leading to their formation and receptor binding remain still poorly understood [48]. While cadherins and amino peptidases frequently emerge as candidates in Cry toxin binding [49], a number of other potential receptors have been proposed including alpha amylases and alpha glycosidases [50,51], prohibitin [52] and alkaline phosphatases [53,54]. The precise role of multiple putative receptors identified for individual toxins is, as yet, unclear.

#### 3.1.2. Non-Three-Domain Cry Toxins

In addition to the major family of three-domain Cry toxins, several other families of unrelated toxins are covered by the Cry nomenclature. Despite a low level of primary sequence identity, which leads them to be classified under distinct primary rankings in the nomenclature, Cry15, Cry23, Cry33, Cry38, Cry45 (parasporin 4), Cry51, Cry60 and Cry64 all show features of the ETX_MTX2 family that includes the Mtx2 protein from Ls and the *Clostridium* epsilon toxin. The latter toxin has an extended beta sheet structure related to aerolysin, a pore-forming toxin produced by the Gram-negative bacterium *Aeromonas hydrophila* and other related species [55,56], and forms beta-barrel pores in target cells [57]. A similar mode of action is also likely for the Cry toxins above.

**Figure 6:**
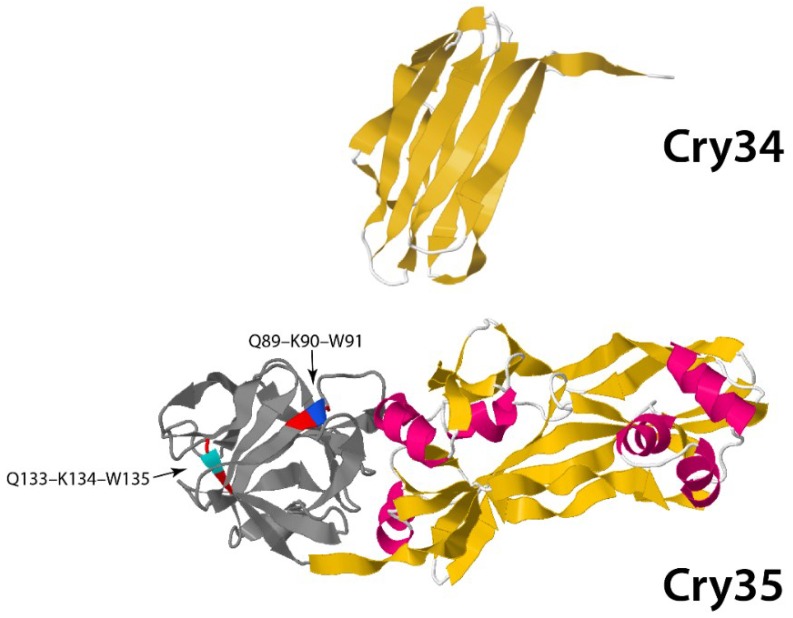
Three-dimensional structure of binary Cry34/Cry35 toxin from PDB accession numbers 4JOX (Cry34) and 4JPO (Cry35) [58]. QxW motifs are depicted as colored residues: red-blue-red and red-cyan-red at positions Q89-K90-W91 and Q133-Q134-W135, respectively, located at the *N*-terminal domain (gray).

Other toxins belong to the Toxin_10 family of proteins and include Cry35 and Cry36 from Bt (in addition to BinA, BinB and Cry49 from Ls). The recently published crystal structures of Cry35Ab1 [58] and BinB [59] show these proteins also to have an aerolysin-like fold. Cry35 has a beta trefoil *N*-terminal domain containing QxW motifs similar to those found in carbohydrate-binding domains in proteins, such as ricin and Mtx1 from Ls (Figure 6).

The rest of the molecule is dominated by an extended beta sheet structure, mirroring that of aerolysin. The crystal structures of Cry45 (Ps4), Cry46 (Ps2) and Cry23 [21,60,61] also show aerolysin folds. Thus, the Toxin_10 group of proteins along with Cry23, Cry45 and Cry46 may also represent beta pore forming toxins, sharing aerolysin as a structural homolog with the ETX_MTX2 family. It is interesting to note that some of the above toxins are able to act alone to cause toxicity (e.g., Cry36), whereas others require a second protein to act as binary toxins (e.g., BinA/BinB, Cry23/Cry37, Cry34/Cry35, Cry48/Cry49) [21,62].

Cry34 is a member of the aerolysin family, which has known interactions with membranes. Its recently published structure [58] (PBD accession number 4JOX) shows a single domain protein with a beta-sandwich conformation and a hydrophobic core (Figure 6). It is interesting to note that, although there is no obvious homology at the level of their amino acid sequences, the Cry34/Cry35 pair show remarkable structural similarity to another binary toxin, Cry23/Cry37 [21]. The precise interactions by which these binary toxins elicit their activity against coleopteran targets remains to be elucidated.

Another non-three-domain protein, Cry22 is reported to show 4 cadherin-like domains and a *C*-terminal region with structural similarities to domain III of the three-domain toxins [21]. No structural data are available for the Cry6 proteins, which show features of the Smc chromosome segregation protein family within their central regions. Studies of the mechanisms of action of these proteins are limited [63,64] and the possible roles of these features are unknown. Cry55 also has no known structural homologs and shows no conserved domains and, as a result, its mode of action also remains unclear.

### 3.2. Cyt Toxins

**Figure 7:**
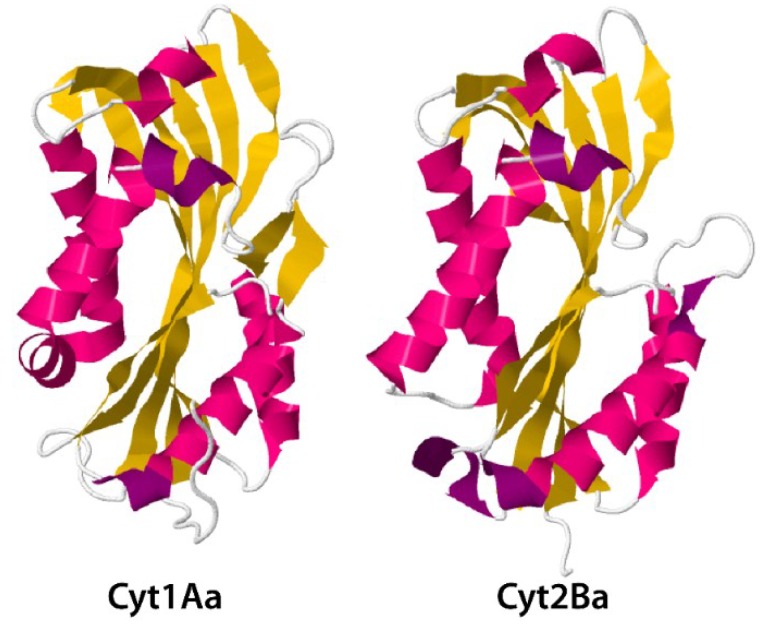
Three-dimensional structure for toxin Cyt1Aa and activated Cyt2Ba monomer from PDB accession numbers 3RON [20] and 2RCI [66], respectively.

Cyt (cytotoxic) proteins, coded for by *cyt* genes, constitute another relevant insecticidal protein family in Bt [65]. In contrast to Cry proteins, Cyt proteins exhibit a general cytolytic (hemolytic) activity *in vitro* and predominantly dipteran specificity *in vivo* [19,21]. The three-dimensional structures of the Cyt1Aa [20] and Cyt2Ba [66] show these proteins to be single domain, three-layer alpha-beta proteins (Figure 7).

The Cyt1Ca protein encoded by the pBtoxis plasmid of Bt subsp. *israelensis* [67] is different in having a further domain with homology to the carbohydrate binding domain of ricin, attached to the *C*-terminal end of the Cyt domain but no larvicidal or hemolytic activity has been observed with this toxin [68]. To date, the Bt Toxin Nomenclature Committee [8] has classified Cyt proteins into three different families (Cyt1, Cyt2 and Cyt3; primary rank) with toxicity mostly against some mosquitoes and black flies [22]. However, some strains of diverse Bt subspecies, e.g., subsp. *morrisoni*, bear *cyt* genes with toxic activity against a wider range of insects, including Diptera, Lepidoptera and Coleoptera [65]. It has also been reported that Cyt2C may be active against nematodes (Rhabditida) and cancer cells [9]. Another interesting feature of Cyt proteins is their ability to synergize the insecticidal activity of other Cry or Vip3 toxins and to reduce levels of insect resistance to some Cry proteins in some insect species [22,23]. For instance, Cyt1Aa toxin is active against *Chrysomela scripta* and inhibits the development of resistance to Cry3Aa [69]. Similarly, Cyt1Aa is able to suppress resistance to Cry4 and Cry11Aa toxins in larvae of laboratory selected *Culex quinquefasciatus* populations [22]. Binding of Cry11Aa to Cyt1Aa facilitates both the oligomerization of Cry11Aa toxin and pore formation, and has been proposed as the mechanism leading to synergism [22]. Moreover, toxins Cyt1Ab and Cyt2Ba from Bt subsp. *medellin* and subsp. *israelensis* enhanced the insecticidal activity of Ls against *Aedes aegypti* and resistant *C. quinquefasciatus* larvae [70]. Cyt1Aa has also been demonstrated to have a synergistic activity, when combined with Mtx1 toxin from Ls, against *C. quinquefasciatus* [71]. Two different modes of action have been proposed for the Cyt group of proteins: one suggests a pore-formation model, whereas the other supports a less specific detergent action mechanism [19,21,22]. For toxins like Cyt1Aa, with a typical cytolysin fold and a specific hemolytic pattern that differs from ionic and non-ionic detergents, a pore-forming mechanism was further suggested [20].

## 4. Secreted Toxins

During the vegetative growth phase of Bt, some strains produce proteins that are secreted into the medium and have been found to have insecticidal properties against a number of insects, extending the overall host range of this bacterium [5,72,73]. The secreted insecticidal proteins constitute two classes that were designated as vegetative insecticidal proteins (Vip) [8,13,14] and secreted insecticidal protein (Sip) [15]. Currently, the Bt Toxin Nomenclature Committee [8] has identified and classified Vip proteins into four different families namely Vip1, Vip2, Vip3 and a novel family of Vip proteins, recently identified and classified as Vip4 by the Bt Toxin Nomenclature Committee [8]. Bt secretable proteins like Vip1, Vip2 and Sip, contain conserved signal peptide sequences that are commonly cleaved before or after the secretion process is completed [13,14,15,21,74]. Vip1 and Vip2 constitute a binary toxin with high insecticidal activity against some coleopteran pests [14] and the sap-sucking insect pest *Aphis gossypii* (Hemiptera) [75]. In contrast, Vip3 proteins are single-chain (not binary) toxins with insecticidal activity against a wide variety of lepidopteran species [13].

### 4.1. Vip1/Vip2 (Binary) Toxins

**Figure 8:**
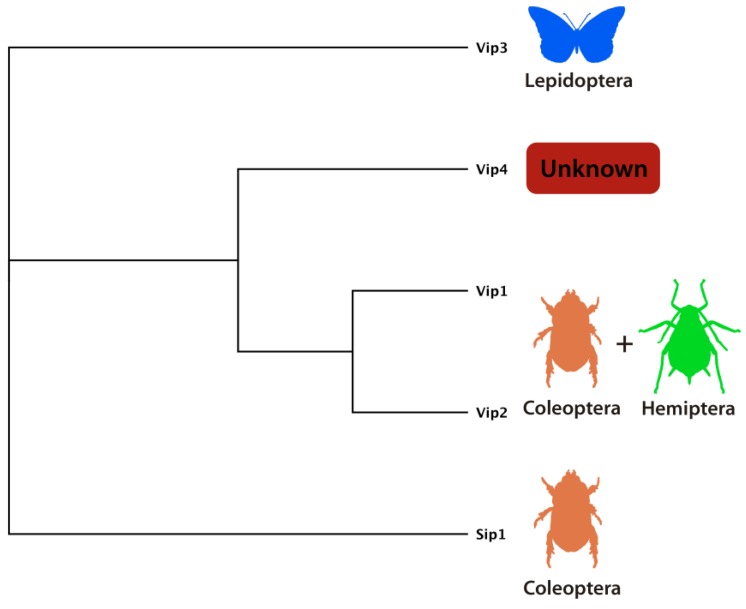
Schematic tree showing the distribution and known host spectrum of different secreted Bt toxins namely Sip1 protein, toxic for some Coleoptera [15], Vip1/Vip2 (binary) toxins active against Coleoptera [14] and Hemiptera [75], Vip4 unknown toxicity and host range [8] and Vip3 toxins active against Lepidoptera [13].

The Vip1 and Vip2 proteins together constitute a binary toxin. The *vip1* and *vip2* genes are co-transcribed from a ~4-kb single operon and encode ~100 and ~50-kDa proteins, respectively [21,76]. They were initially discovered from Bc strain AB78 and Bt in the 1990s [14,21,74,77]. Vip1 and Vip2 contain typical *Bacillus* *N*-terminal signal peptide sequences and Vip1 is *N*-terminally processed after secretion into a smaller ~80-kDa mature protein. Vip1/Vip2 showed toxic activity against some coleopteran larvae (e.g., *Diabrotica* spp.) and the sap-sucking insect pest *Aphis gossypii* (Figure 8) [14,21,75]. Their homology to other bacterial binary toxins suggests that Vip1 and Vip2 form typical A+B type binary toxins, where Vip2 is the cytotoxic A-domain and Vip1 the receptor-binding domain responsible of the translocation of the cytotoxic Vip2 into the host cell [21,77]. Vip2 exhibits sequence and structural homology with the enzymatic domain of toxin CdtA from *Clostridium difficile* and the iota-toxin domain Ia from *C. perfringens*, both possessing ADP-ribosyltransferase activity that targets actin, inducing cytoskeletal disorders and cell death [77,78]. The crystal structure of Vip2 from Bc in apo and NAD^+^ complexed form is consistent with this mechanism of action [78]. The proposed mechanism of action involves the proteolytic activation of the cell-binding B precursor (Vip1) and its monomeric interaction with cell surface receptor(s) followed by formation of homoheptamers that subsequently translocate the A (Vip2) toxic component into the cytoplasm through acid endosomes [77]. Once inside the cytoplasm, the A component destroys filamentous actin, likely by mono-ADP-ribosylation of the Arg177 residue of G-actin, blocking its polymerization and leading to cell death by cytoskeletal disarrangement [74,79].

### 4.2. Vip3 Proteins

The first *vip3* genes were originally cloned from total DNA libraries of Bt strains AB88 and AB424 and were designated as *vip3Aa1* and *vip3Ab1* [13], respectively. They encode 791-aa proteins (88.5-kDa) and show no homology to any other known insecticidal protein [13,21].

Vip3A proteins exhibit insecticidal activity against a wide variety of lepidopterans and, interestingly, certain species less susceptible to some Cry1A proteins (e.g., *Agrotis ipsilon*, *Spodoptera exigua* and *S. frugiperda*) [13,80]. To date, there are three different subfamilies of Vip3 proteins that were designated Vip3A [13], Vip3B [81] and Vip3C [82] (Figure 8). In contrast with Vip1 and Vip2, Vip3 proteins contain conserved but non-typical signal peptide sequences that are not processed during secretion and are present in the secreted mature peptide [13,21,83]. Treatment of Vip3Aa1 with midgut fluids from susceptible insects or trypsin releases four major protein products with molecular weights of approximately 22, 33, 45, and 66-kDa [84]. The 66-kDa fragment is separated from the 22-kDa *N*-terminal portion and is known as the activated “trypsin resistant core”. This processed portion of Vip3 proteins, which is the major polypeptide fraction that retains toxicity, may vary in size from 62 to 66-kDa [84,85], and remains highly conserved between different Vip3 proteins. When this activated fragment was cloned and expressed in *E. coli*, no toxicity was observed against insect species previously found to be susceptible, suggesting that the 22-kDa *N*-terminal portion is necessary for toxicity or folding [13,21,83]. Preliminary analysis of the structure of the Vip3Ag4 protoxin by electron microscopy, strongly suggests that molecules of the protein may be grouped together forming a tetrameric structure [86]. While there is evidence that Vip3 proteins act by pore formation [87], the mode of action of Vip3 proteins remains poorly understood. A proposed mechanism suggests that activated Vip3A toxin binds to the midgut epithelial cells of susceptible insects causing their lysis, gut paralysis and larval death [21,88]. It has been suggested that this mode of action differs from that of some Cry1A toxins, at least with respect to receptor binding and ion channel properties. Indeed, Vip and Cry proteins do not compete for binding sites in the lepidopterans *Manduca sexta* and *S. frugiperda* [87,89]. Additionally, extensive *in vitro* and *in vivo* studies have shown very low levels of cross-resistance between Vip3A and Cry1 proteins [85,90,91]. These Vip3 properties open the possibility to use Bt-based biopesticides for a greater number of pests and for the management of emerging insect resistance events to Bt Cry proteins [89,92,93].

### 4.3. Vip4 Protein

**Figure 9:**
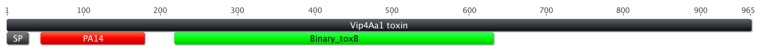
Predicted features and conserved domains found in Vip4Aa1 toxin. SP: predicted signal peptide (residues 1–28), PA14: conserved domain (residues 45–179), Binary_toxB: conserved domain (residues 218–631) commonly found in Vip1Aa1 proteins and present in novel Vip4Aa1 toxin.

A novel protein has been identified by Sun *et al.* (2010) and designated Vip4Aa1 (Figure 9) [8]. The *vip4Aa1* gene is 2895 bp long with a deduced amino acid sequence of 965 residues and predicted molecular weight of ~108-kDa. Its predicted protein sequence exhibited 34% and 65% pairwise identity to Vip1Aa1 protein and the Iota toxin Ib from Bc, respectively. Protein sequence analysis detected a putative signal peptide sequence plus two conserved domains: an anthrax protective antigen PA14 domain (InterPro family IPR011658) and a bacterial Binary_ToxB exotoxin domain (InterPro family IPR003896) commonly found in binary Vip1 and other related bacterial toxins (Figure 9). The insecticidal properties (activity and host range) of this protein remain unknown, although Vip4Aa1 seems to be phylogenetically more closely related to Vip1 proteins (Figure 8) than to the other two Vip groups (Vip2 and Vip3) [8]. Since it resembles the B component of a binary toxin, it is possible that Vip4 interacts with an, as yet, unidentified A component, perhaps a Vip2, to elicit toxicity. A further possibility is that it is a B-component that has lost its A-component and can no longer confer toxicity. Analysis of the genome of strain Sbt016, from which Vip4 was identified, may shed some light on these questions.

### 4.4. Sip Toxin

The secreted insecticidal protein (Sip) constitutes the first and only member of a Bt insecticidal family of secreted proteins with demonstrated toxicity against coleopteran larvae. Sip protein was initially obtained from culture supernatants of the Bt strain EG2158 and was designated as Sip1Aa1 [15]. The *sip1Aa1* gene is 1104 bp long and encodes a protein of 367 amino acids and ~41-kDa. Sip1Aa1 exhibits typical predicted Gram-positive consensus secretion signal 30 amino acids long. However, the protein was found *N*-terminally processed, with its first 43 amino acids eliminated by active proteases present in the culture medium. It shows low but significant similarity to the 36-kDa Mtx3 mosquitocidal toxin (a member of the ETX_MTX2 family of toxins) from Ls. This homology strongly suggests that Sip1Aa1 toxicity may be caused by pore formation, but its mode of action remains unknown [15]. Sip1Aa1 is lethal for *Leptinotarsa decemlineata* (Coleoptera: Chrysomelidae) and inhibits growth of *Diabrotica undecimpunctata howardi* (Coleoptera: Chrysomelidae) and *D. virgifera virgifera* [15]. Although Sip1Aa1 is the only protein reported to date with published insecticidal activity against these insects, several other homologs with at least 90% pairwise identity have been published as hypothetical proteins in public databases (e.g., GenBank accession number WP_000875422).

## 5. Other Potential Insecticidal Toxins

Bt also synthesizes other proteins, the sequences and conserved domains of which suggest that they may be toxic agents but which have received little investigation. In addition, some strains produce the non-proteinaceous β-exotoxin, which shows broad-spectrum activity.

### 5.1. A 41.9-kDa Protein

Several strains of Bt and Bc encode a protein of approximately 42-kDa that has been called the 41.9-kDa protein (e.g., the Bc protein, accession number WP_000727409). These putative 41.9-kDa insecticidal toxins show some similarity to BinA and BinB, respectively, the two components of the Bin (binary toxin) from Ls. Their sequences revealed two conserved domains, a ricin B-like lectin (InterPro family IPR000772) and a *Bacillus* Toxin_10 crystal toxin conserved domain (InterPro family IPR008872), commonly found in other Ls and Bt toxins. To date, only one homolog protein isolated from Bt has been tested against a limited range of insects and was found to be non-toxic to a number of lepidopteran species and the green peach aphid *Myzus persicae* (Hemiptera) [94]. This may indicate that the protein has another target, that it requires another protein for its activity acting as a binary toxin or that it is not, in fact, a toxin.

### 5.2. Sphaericolysins and Alveolysins

Proteins related to cholesterol-dependent cytolysins, such as sphaericolysin from Ls and alveolysins are present in some Bt strains (e.g., sequence accession number ZP_04117355 from *B. thuringiensis* subsp. *kurstaki*) [16]. On injection, a member of this family, sphaericolysin from Ls, has activities against the cockroach *Blattella germanica* and the lepidopteran *Spodoptera litura* [95]. The very high level of sequence conservation in this family [96] makes it likely that the homologs from Bt strains will also show toxic activity.

### 5.3. Beta Exotoxins

Some Bt strains are capable of producing non-proteinaceous, thermostable and secretable secondary metabolites exhibiting non-specific toxic activity not only against a wide range of insects but also against mammals [97,98]. These secondary metabolites, commonly known as β-exotoxins (e.g., thuringiensin), are low molecular weight (700-Da) analogues of the nucleotide adenine. The production of a related beta-exotoxin type II by some strains has also been reported and this may be the uracil analog of thuringiensin [99]. Beta exotoxins cause their toxic effect by inhibition of DNA-dependent RNA polymerase [100], probably the reason why they may also affect mammals [101]. Beta exotoxin production is more prevalent in some serovars than others [102] and the absence of β-exotoxins is a requirement for Bt formulations in Europe, the US and Canada [103].

### 5.4. Enhancin-Like Proteins

Enhancin-like proteins exhibit 20%–30% pairwise similarity to viral enhancin proteins (metalloproteases), which favour viral infections by degrading the mucin of the protective peritrophic matrix. One of these, commonly known as Bel enhancin, has been reported to be effective in enhancing the toxicity of Cry1Ac against *Helicoverpa armigera* [104], whereas an identical enhancin-like protein, sharing 100% of pairwise identity with Bel enhancin, showed no significant increase in toxicity against *S. exigua* and *Trichoplusia ni* when combined with Cry9Ea toxin [105].

### 5.5. P19 and P20 Helper Proteins

Crystal proteins accumulate in the cells as parasporal inclusions and can account for up to 25% of the sporulated cell dry weight [106]. Many of the Cry proteins, for example the 125- to 140-kDa protoxin forms of three-domain toxins, are capable of directing their own crystallization. However, other three-domain toxins that lack the extended *C*-terminal region in their protoxins (e.g., Cry11Aa), may require the collaboration of at least one of two accessory helper proteins (P19 and P20) [67,106,107] for the stable production of parasporal crystals. These helper proteins may also have a more complex role, because P20 enhances the expression and crystallization of Cry1Ac in plasmid-negative (acrystalliferous) Bt strains [108] and synergizes the toxic activity of Cry11A against third-instar larvae of *A. aegypti* [109]. P20 also appears to have a role in stabilizing Cyt1Aa [106] and increases Cyt production in Bt [68,110,111] and circumvents problems with Cyt expression in *E. coli* [112].

## 6. Mechanisms of Toxin Evolution

Bt thus synthesizes a vast number of protein toxins with activity against a wide range of organisms in nature, including not only a broad range of insect orders but also nematodes, a human-pathogenic protozoan, animal and human parasites plus different human-cancer cell lines [5,9,11,26,30,113]. Recently, a Bt strain exhibiting molluscicidal activity against the snail pest *Cernuella virgata* has been reported, although the molecule causing toxicity remains unknown [114]. The reasons driving such wide toxin diversification are not well understood. Perhaps, some of the most important factors are three physiological conditions that vary greatly among insects: the gut pH, midgut proteases and toxin receptors [21,25]. Such factors probably exert selective pressure in toxin coding genes, forcing their evolution to adapt and overcome current and novel host defenses. For example, most of the active toxins against lepidopterans must be solubilized in the alkaline midgut and activated by proteolytic digestion of serine proteases, absent from the acidic coleopteran and hemipteran midguts. In turn, these insect guts possess mainly cysteine and aspartic proteases [21,25,115]. Interaction of insecticidal toxins with specific receptors on the gut epithelium is also a key factor in toxin evolution. For instance, resistance events against the most used Cry toxins are frequently attributed to alterations in toxin-receptor interactions [21,116]. Finally, the genomic sequence itself and the organization of toxin-coding genes in the genome may also contribute to toxin evolution. Most Bt strains bear large plasmids containing their toxin-coding gene repertoires [21,67,106,117,118]. The current opinion is that these plasmids are not self-mobilizable since there is not still enough evidence on their transfer mechanism except for a transfer-mediated conjugation system, reported for the Bt pX016 plasmid that may be able to mobilize toxin-coding plasmids between Bt strains [21,119] and from Bt to Ls [120]. Transfer of plasmids in infected insect larvae has also been shown in e.g., the lepidopterans *Galleria mellonella* and *Spodoptera littoralis* [121] in the soil, in other larvae and on leaf surfaces [122,123,124]. Transconjugant strains combining plasmids have been produced and commercialized (*Foil* for use against Lepidoptera and Coleoptera and *Condor* for use against spruce budworm and gypsy moth: produced by Ecogen Inc., Langhorne, PA, USA) and indicate that the plasmids from different strains can be compatible. The movement of plasmids in nature, may account for the discovery of *cry* toxin genes, related to those of Bt, in Ls [62], *Paenibacillus popilliae* (e.g., [125]), *Paenibacillus lentimorbus* [126] and *Clostridium bifermentans* serovar. *malaysia* [127] as well as the presence of *cyt*-like genes in *Erwinia* [128] and *Dickeya dadantii* [129]. The latter bacterium is a pathogen both of plants and of the pea aphid *Acyrthosiphon pisum* and it appears that induction of *cyt* expression under conditions similar to those in phloem may be a factor in the aphid pathogenicity of the bacterium. In addition, the mobilization of plasmids between Bt strains might explain why they contain exact copies of a given Cry protein-coding gene and are distributed among geographically distinct Bt strains, for instance, the Bt strain Na205-3 (isolated from Navarra, Spain) [130] shares some, but not all, plasmids containing Bt toxin genes with Bt strain IS5056 (isolated from Biebrza, Poland) [131]. This phenomenon has also been observed among holotype *vip3* gene variants (e.g., *vip3C*) [82] and may explain the isolation of other identical or nearly identical Vip3 protein-coding genes in more than 50 strains isolated worldwide [8]. Both *cry* and *vip* genes (and probably the *cyt* genes) are subjected to adaptive evolutionary forces that drive their evolution and specificity [82,132,133]. Phylogenetic studies suggest that genes encoding three-domain Cry proteins evolved from a common ancestor and that their diversity may have be enhanced by sequence divergence and homologous recombination [21]. Recombination of related but distinct toxin sequences seems to be a significant process in toxin evolution. Within the family of so-called three-domain Cry toxins, there are significant regions of identity [17], particularly in five conserved sequence blocks in the region of the active toxin [1] and throughout the *C*-terminal region of the large (125–140-kDa) protoxins [134], it is postulated that this may facilitate recombination between toxin genes. The occurrence of such rearrangements is indicated by the apparently different rates of evolution for each of the three domains [38] with the pore-forming domain I evolving most slowly and domain II, which has a role in receptor binding, evolving most quickly. Rearrangement and domain shuffling of this type has been suggested as a source of dual activity (lepidopteran/coleopteran active) for toxins [135]. Further evolution of the toxins by the selection of natural mutants is likely to follow large-scale rearrangements and key residues in Cry toxin domains II and III appear to be under particular adaptive pressure [132] as does the *C*-terminus of the Vip3 protein [133]. It is interesting to note that phylogenetic trees based on the three domains, produce different families of toxins from those using full protoxin sequences [17,38,135]. It is possible that the location of toxin genes on plasmids may allow a higher rate of recombination due to the greater proximity of individual genes (relative to genes scattered around the main genome). Recombination events during toxin evolution may also be the source of sequences on the toxin-coding plasmid pBtoxis, of Bt serovar. *israelensis* that appear to be remnant fragments of toxin genes [67]. Gonzales and Carlton demonstrated the potential of this plasmid to rearrange and recombine with other plasmids in different ways, when subjected to growth at an elevated temperature of 42 °C. For instance, recombination of pBtoxis (which they referred to as the 75-MDa plasmid) was observed with a 68-MDa (~110 kb) plasmid to create new 63-MDa (~105 kb) and 80-MDa (~135 kb) plasmids [136]. The apparent absence of *cry4Aa* and *cry10Aa* genes from the toxin coding plasmid related to pBtoxis in the Tunisian isolate BUPM97 of Bt serovar. *israelensis* [137] may be explained by such rearrangements or could represent a plasmid that had never acquired these genes while plasmid pBTHD789-3 from Bt israelensis strain HD789 is also similar to pBtoxis but carries an additional Cry4B gene and two Cry60 genes [138]. Finally, many toxin-coding genes have been found flanked by sequences coding for putative transposons and transposases [21,67]. This may indicate that these genes can be acquired independently and represent a further source of toxin evolution. The vestigial toxin gene remnants of the pBtoxis plasmid, especially those containing nearby transposons sequences, may represent evidence of recombination or transposition events [21,67]. Gene duplication is evident in Ls where two identical copies of the genes encoding the mosquitocidal Bin toxin are encoded; one copy in the genome and one copy on the large pBsph plasmid [139,140]. Gene duplication, followed by sequence divergence, may also lead to small-scale diversification of toxins active against the same target. Different members of the same Cry toxin family in individual isolates (e.g., Cry1Aa, Cry1Ab & Cry1Ac and Cry2Aa and Cry2Ab in Bt serovar. *kurstaki* HD1 [141] may be a result of such events and the presence in strains of multiple toxins from a single first-level Cry family is common [141,142]. The resulting toxins may, however, share receptors [143,144] and in some instances, may interact antagonistically [145,146].

## 7. Novel Toxins and Next-Generation Sequencing (NGS) Technologies

Since the first *B. thuringiensis* insecticidal genes were discovered, the search for other genes encoding novel proteins with promising insecticidal properties and specificities has continued. Such screenings were carried out mainly with polymerase chain reaction (PCR) or a combination of PCR and restriction fragment length polymorphism (PCR-RFLP) approaches [147,148,149,150,151,152]. However, these PCR-based techniques are slow, laborious and limited, because they mainly identified alleles of previously described genes; these new genes usually displayed just a small number of synonymous or non-synonymous substitutions, and encoded identical or slightly different proteins compared to previously described alleles, likely having very similar toxicity and host range. In addition, since these techniques yield only a fraction of the gene sequence, time-consuming PCR walking strategies or construction of genomic libraries and screening are needed to obtain a single full-length coding sequence suitable for cloning.

Finding novel proteins is particularly important to manage the increasing occurrence of resistance to Bt-based insecticides or transgenic Bt plants [116,153,154]. Today, next-generation sequencing (NGS) technologies [155,156,157,158,159] are available as novel and useful tools for the discovery of previously unrecognized insecticidal-toxin genes that would otherwise be difficult to identify. NGS technologies provide a fast and reliable framework to obtain complete genomic sequences, and offer excellent cost-benefit ratios since complete genome sequences can be obtained for less than 0.1–1 euro per kilobase. This cost may be reduced even further if only raw sequence production is outsourced and the researcher completes the assembly steps. Sequences can readily be assembled in contigs using a regular desktop computer [160] with *de novo* assembly tools bundled in bioinformatic software, such as CLC Genomics Workbench or Geneious Pro (both commercial software), or using diverse open source assemblers freely available in the BioLinux distribution [161], among other alternatives [162]. Insecticidal gene prediction and annotation could be easily accomplished using the NCBI Blast tools [163], using customized (insecticidal) BLAST databases [94,130,164] or the BtToxin_Scanner [165]. Once the full-length toxin-coding sequence (or sequences) is identified, it can be easily amplified by PCR and cloned for protein expression, in the host system of choice.

While mass sequencing programs offer huge potential for the discovery of new toxin sequences, the number and diversity of toxins discovered will present real challenges to the toxin nomenclature system that has served the Bt community well for many years. Such challenges include the sheer numbers of new variant toxins that may overwhelm the system as well as nomenclature difficulties within the present system. For example, the current nomenclature may contain two proteins with different secondary ranks (e.g., Cry5A and Cry5E) when a new variant sharing >78% identity with both is discovered. Placing such new variants in the nomenclature will present difficulties as more toxins emerge and solutions to this issue will be needed.

## 8. Concluding Remarks

*Bacillus thuringiensis* (Bt) is a Gram-positive, spore-forming bacterium that synthesizes an extraordinary diversity of insecticidal proteins and has demonstrated its potential and safety as a biocontrol agent over more than five decades. There is now an extensive literature on a wide range of Bt-related topics from its natural ecology to its mode of action. A large number of toxins, active against a wide range of invertebrates have been described along with a number of proteins with activities against cancer cells in culture. The evolution and diversification of these toxins is likely to be driven by an arms race between pathogen and target and to be facilitated by recombination events between related genes, transposition and plasmid transfer. The insecticidal proteins include both crystal and secreted proteins highly toxic against a wide range of invertebrate species [5,6,18,26] and our understanding of their modes of action and structural biology are increasing, as described in this review. Several natural Bt strains have been incorporated successfully in the production of sprayable Bt-based bioinsecticides wherein the active ingredient is a mixture of spores and protein crystals.

The wide diversity of insecticidal proteins produced by this bacterium suggest that their encoding genes are affected by strong selective evolutionary pressures [18,105,106] leading to the expansion of the range of targets and turning Bt into a rich source of proteins with toxic activities against insects and other organisms. The innovations of NGS technologies are likely to increase our arsenal of toxins still further and with increased efficiency. This expansion in toxin discovery is also likely to put the toxin nomenclature system under increasing pressure and this may require novel strategies to keep pace with the new technology.

## Abbreviations

Bc: *Bacillus cereus*
Bt: *Bacillus thuringiensis*
Ls: *Lysinibacillus sphaericus*
